# Modeling and predicting growth and growth boundary of *Bacillus cereus s.l.* from phylogroups II, IV, V, and VI in starchy foods at or below 12°C

**DOI:** 10.3389/fmicb.2025.1531014

**Published:** 2025-04-30

**Authors:** Veronica Martinez-Rios, Resadije Idrizi, Paw Dalgaard, Lisbeth Truelstrup Hansen, Tina Beck Hansen

**Affiliations:** The National Food Institute (DTU Food), Technical University of Denmark, Kongens Lyngby, Denmark

**Keywords:** extended shelf life, food safety, *panC* groups, ready-to-eat, spore-formers, chilled storage, ready-to-cook

## Abstract

Pathogenic *Bacillus cereus s.l.* can survive cooking of starchy foods and grow at chilled storage temperatures, highlighting foods with extended chilled shelf life as a risk factor. Some food administrations encourage use of predictive microbiology to support decisions of safe shelf lives. Therefore, the present study embarked on identifying a model from literature and/or expanding an existing model to enable accurate predictions of growth and no-growth responses of relevant *B. cereus s.l.* in starchy ready-to-eat and ready-to-cook foods when stored at temperatures at or below 12°C. The study focused on isolates belonging to psychrotolerant or mesophilic-psychrotolerant intermediary thermotypes in *panC*-groups II, IV, V, or VI and generated data for growth kinetics for various pH (4.8–7.8), a_w_ (0.935–0.999) and storage temperatures (6.0–11.7°C) in 42 starchy foods (bulgur, couscous, pasta, potatoes, rice) and eight composite foods containing at least one starchy ingredient. Using 21 of the growth kinetics obtained for starchy foods, the five best performing of 10 available growth models were selected for improvement by product calibration and/or expansion with terms to consider the effect of interactions between temperature, pH and a_w_. Of 410 updated models, nine showed promising performance and were evaluated using the remaining 21 growth kinetics obtained in starchy foods. Two models could be considered validated for these products with *B_f_*/*A_f_* –values of 0.87/1.21 and 1.01/1.32, respectively. Both models provided ≥75% correct predictions of the growth/no-growth responses and did not provide any fail-dangerous predictions. Further evaluation of these models for predictions of maximum specific growth rates (*μ_max_*, h^−1^) and growth/no-growth responses for a broader range of starchy foods used 33 challenge tests from the scientific literature and eight challenge tests from the present study, and remarkably showed that the performance of both models was poor for composite protein-rich starchy foods with *B_f_ –*values ≤0.64 and *A_f_* –values ≥1.96, meaning these models should not be used for such products as μ_max_ might be under-predicted creating unsafe situations. However, for other starchy foods, one of the validated models was found to be acceptable on the safe side with *B_f_* – and *A_f_* –values of 1.34 and 1.57, respectively.

## 1 Introduction

Several species from the *Bacillus cereus* group, also known as *Bacillus cereus sensu lato*, are known as significant foodborne hazards warranting food safety management in processed ready-to-eat and ready-to-cook foods (Daelman et al., 2013a,b,c; Webb et al., 2019). *B. cereus s.l.* are sporeformers and their endospores are widely distributed in the environment from where they can contaminate many kinds of food raw materials (Vos et al., 2011). Recently, a systematic review pointed to cereals, beans, and vegetables as raw materials with presence of *B. cereus s.l.* in 37–45% of samples (Rahnama et al., 2022). As the endospores are highly heat-tolerant, they can survive cooking of food (den Besten et al., 2018; Le Marc et al., 2022; Luu-Thi et al., 2014), meaning *B. cereus s.l.* are also readily detected from heat-treated foods (Rosenquist et al., 2005; Samapundo et al., 2011; Turner et al., 2006).

*B. cereus s.l.* have been found to cause foodborne outbreaks, with 413 strong-evidence outbreaks reported to EFSA’s Zoonoses database during the eight-year period from 2007 to 2014 (EFSA BIOHAZ Panel, 2016). In 2022 alone, 306 outbreaks caused by *B. cereus s.l.* were registered in EU, which increased to 474 in 2023 indicating an increasing number of reported outbreaks (EFSA and ECDC, 2023, 2024). Bakery products, cereal products (including rice and seeds), and mixed foods (e.g., paella, risotto and curries) are typical foods associated with outbreaks caused by *B. cereus s.l.* (EFSA BIOHAZ Panel, 2016). Thus, starchy foods and/or composite foods containing starchy ingredients are significant sources for foodborne outbreaks related to *B. cereus s.l.* Rice-based products and starchy foods, together with vegetable-based dishes have also been highlighted as important sources of *B. cereus s.l.* outbreaks in European large scale catering (Osimani et al., 2018). A recent analysis, from the Zhejiang Province in China, found that *B. cereus s.l.* caused 5.6% of all registered outbreaks from 2010 to 2020 (Chen et al., 2022). Most of the Chinese *B. cereus s.l.* outbreaks were traced back to cereals or flour products confirming that heat-treated foods containing starch should be considered as particularly important sources of *B. cereus s.l.*, which will require implementation of food safety management for mitigation of the risk.

The risk of outbreaks occurring is mainly associated with growth of *B. cereus s.l.* in foods where spores have survived the heat-treatment to go on to germinate and grow, e.g., during improper cooling or holding of foods for too long at ambient temperatures (Osimani et al., 2018). Moreover, since some *B. cereus s.l.* sub-groups grow at chilled temperatures, cooked products with extended chilled shelf life also represent a risk factor (Carlin et al., 2013; Daelman et al., 2013a; Webb et al., 2019). Foods, where product characteristics and storage temperature will allow growth of *B. cereus s.l.* sub-groups to more than 10^5^ cfu/g before consumption, should be considered hazardous as cells or spores may cause toxico-infection and/or formation of cereulide that can cause intoxication in consumers (EFSA BIOHAZ Panel, 2016; Webb et al., 2019). The ability to grow at chilled storage temperatures is confined to specific *B. cereus s.l.* sub-groups and partial sequencing of the pantoate beta-alanine ligase (*panC*) gene has been widely used to divide isolates into phylogenetic sub-groups with different ability to grow at low temperatures (Carroll et al., 2022; Fiedoruk et al., 2017; Guinebretière et al., 2008).

Chilled storage has been specified as the keeping of foods at temperatures of 8°C or below in many European countries. However, European Food authorities, including the Danish Veterinary and Food Administration, are considering allowing producers of food more flexibility in the setting of storage temperatures and encourage their use of predictive food microbiology to establish safe shelf lives that correspond to the new storage temperature (EFSA, 2015; Ministeriet for Fødevarer, Landbrug og Fiskeri, 2013). Some mathematical models are available to predict growth of *B. cereus* depending on product storage temperature, pH and salt or water activity (a_w_) (Carlin et al., 2013; Sutherland et al., 1996; Zwietering et al., 1996). Nevertheless, few validation studies have documented the ability of these models to accurately predict growth of *B. cereus s.l.* sub-groups in different types of chilled foods. This is important because available growth models were developed using liquid laboratory broth or milk and it is known also for other bacteria that growth rates in liquid substrates may differ from those in food products with similar temperature, pH and a_w_. Therefore, available *B. cereus s.l.* growth models may need to be calibrated to provide realistic growth rate predictions for starchy foods (Buss da Silva et al., 2017; Koukou et al., 2021).

The present study focused on *B. cereus s.l.* isolates able to grow at or below 12°C and belonging to psychrotolerant or mesophilic-psychrotolerant intermediary thermotypes in *panC*-groups II, IV, V or VI. The objective was to identify a model from the literature and/or expand an existing model so that it can accurately predict growth and no-growth responses of relevant *B. cereus s.l.* sub-groups in starchy ready-to-eat and ready-to-cook foods when stored at temperatures at or below 12°C. Firstly, data for growth kinetics, product characteristics and storage temperatures were generated in three series of challenge tests. Secondly, a part of these data was used to select the best performing available growth models. Thirdly, selected models were product calibrated and/or expanded with terms to consider the effect of interactions between their factors (temperature, pH and a_w_). Finally, the performance of the most suitable models was evaluated using a different part of the generated data as well as data from studies available in the scientific literature.

## 2 Materials and methods

### 2.1 New growth responses and product characteristics generated from challenge tests

#### 2.1.1 Isolates, sporulation and stock of spores

Ten *B. cereus s.l.* isolates were selected for new challenge tests performed as part of the present study (Table 1). Isolates included two from diarrheal outbreaks, six from foods, one from environment and the type strain (Table 1). Stock cultures (−80°C) were grown (30°C, 24 h) in Brain Heart Infusion broth (BHI) (CM1135, Oxoid, Basingstoke, UK) to obtain vegetative cells. Spore stocks were then prepared from these using Nutrient Agar (CM0003, Oxoid) supplemented with Manganese sulfate (M2643, Sigma, Darmstadt, Germany) (NAMS agar) (30°C, 3 days) as described by Beuchat et al. (1997). Spores were harvested using a few modifications of the Beuchat et al. (1997) method. Briefly, 5 mL of saline (0.85% NaCl, 1.06404.1000, Supelco, Darmstadt, Germany) was deposited onto the surface of each agar plate which was gently rubbed with a sterile L-shaped drigalski spatula to release the colony material from the agar surface into the saline obtaining a suspension of spores and vegetative cells. The suspension was filtered through sterile glass wool to remove debris originating from the agar surface, centrifuged at 2,600 × *g* (5°C) for 20 min, and the supernatant discarded. The resulting pellet was suspended in 50 mL of saline and washed twice by centrifugation at 5°C using 6,000 × *g* for 10 min. The final pellet was suspended in 10 mL saline (spore stock) and stored at 6 ± 0.5°C for use in subsequent challenge tests. The concentration of spores in each spore stock was determined by spread-plating 100 μL of appropriate serial dilutions (in 0.85% NaCl) of heat-treated spore stocks on Tryptone Soya Agar (TSA) (CM131B, Oxoid) (30°C, 24 h). The heat-treatment (5 min, 80°C, Beuchat et al., 1997) was conducted on 1-mL aliquots of 100× diluted spore stocks in a heating block (Eppendorf Thermomixer comfort, Eppendorf Nordic A/S, Hørsholm, Denmark).

**Table 1:** *Bacillus cereus sensu lato* isolates used for challenge testing in the present study.

Isolate	Origin	Phylogenetic (*panC*) group^a^	References
RIVM BC120	Diarrheal outbreak	II	Provided by INRA, Avignon, France (Carlin et al., 2013)
NVH 0861–00	Diarrheal outbreak	II	Provided by INRA, Avignon, France (Carlin et al., 2013)
C262	Lasagna	II	DTU Food strain collection (Klein, 2019)
ATCC 14579	Type strain of *B. cereus*	IV	Frankland and Frankland (1887)
C218	Ready-to-cook dish^b^	IV	DTU Food strain collection (Klein, 2019)
T101	White organic rice	IV	DTU Food strain collection (Samadi, 2020)
C246	Vegetable lasagna	V	DTU Food strain collection (Klein, 2019)
T126	Brown organic whole grain rice	V	DTU Food strain collection (Samadi, 2020)
ADRIA I21	Food	VI	Provided by INRA, Avignon, France (Carlin et al., 2013)
KBAB4	Environment	VI	Provided by INRA, Avignon, France (Carlin et al., 2013)

a Based on Guinebretière et al. (2008).

b Pork chops with mashed potatoes, sauce and mushrooms.

#### 2.1.2 Experimental design and challenge tests with single component starchy or composite foods to generate growth responses

A compiled dataset including seven preliminary challenge tests and 35 challenge tests (total n = 42), planned by using a statistical design of experiment (DOE), were split into two sets of 21 challenge tests. One set (*n* = 21, Table 2) was used to evaluate available growth models and then product calibrating these models with or without including a term for the inhibiting effect of interactions between product characteristics (see sections 2.2 and 2.4). The other set (*n* = 21, Table 3) was then used to select the best performing models (see section 2.5).

**Table 2:** Product characteristics, storage conditions and estimates of maximum specific growth rates (*μ_max_*) for challenge tests with single starchy foods used for the evaluation and updating of *Bacillus cereus sensu lato* growth models from literature.

Exp. no.	Cooked food	Isolate	Group^a^	Measured characteristics^b^						Temp.^c^ (°C)		*μ_max_* (h^−1^)^b^	
				pH		% WPS		a_w_					
20	Bulgur	ADRIA I21	VI	6.8	(<0.1)	6.8	(0.06)	0.958	(0.002)	11.7	(0.2)	0.041	(0.002)
24	Bulgur	KBAB4	VI	5.1	(<0.1)	6.0	(0.52)	0.957	(0.001)	10.0	(0.5)	NG^d^	
10	Couscous	NVH 0861–00	II	6.4	(0.1)	3.3	(0.02)	0.983	(0.001)	6.9	(0.2)	0.016	(0.012)
6	Couscous	T101	IV	6.4	(0.1)	3.3	(0.02)	0.981	(<0.001)	11.5	(0.1)	0.109	(0.041)
12	Couscous	C246	V	6.4	(<0.1)	9.0	(0.23)	0.935	(0.002)	11.2	(0.2)	NG	
Pre	Mashed potato	RIVM BC120	II	6.2	(<0.1)	0.06	(<0.01)	0.999	(<0.001)	7.7	(0.1)	0.037	(0.005)
18	Mashed potato	ATCC 14579	IV	5.9	(<0.1)	1.4	(0.10)	0.992	(<0.001)	10.8	(0.6)	0.076	(0.031)
22	Mashed potato	KBAB4	VI	6.2	(<0.1)	0.06	(<0.01)	0.999	(<0.001)	7.7	(0.1)	0.066	(0.001)
32	Pasta	RIVM BC120	II	6.1	(<0.1)	5.5	(0.22)	0.962	(0.002)	10.5	(0.4)	0.036	(0.003)
34	Pasta	NVH 0861–00	II	5.1	(<0.1)	3.0	(0.16)	0.978	(0.001)	7.5	(0.4)	NG	
17	Pasta	C218	IV	6.0	(<0.1)	0.03	(<0.01)	0.995	(0.001)	11.0	(0.1)	0.064	(0.002)
14	Pasta	T126	V	4.8	(<0.1)	3.0	(0.33)	0.978	(0.001)	11.0	(0.1)	NG	
31	Pasta	T126	V	6.1	(<0.1)	4.0	(0.28)	0.970	(0.002)	7.5	(0.4)	NG	
35	Pasta	KBAB4	VI	6.5	(<0.1)	3.7	(0.27)	0.974	(0.001)	10.5	(0.4)	0.074	(0.002)
28	Rice	NVH 0861–00	II	6.6	(<0.1)	7.1	(0.15)	0.956	(0.003)	6.6	(0.3)	NG	
27	Rice	NVH 0861–00	II	5.5	(<0.1)	5.7	(0.1)	0.964	(0.001)	10.0	(0.5)	NG	
11	Rice	T101	IV	6.4	(<0.1)	3.4	(0.06)	0.981	(<0.001)	11.5	(0.1)	0.106	(0.043)
25	Rice	T101	IV	7.8	(0.1)	0.02	(<0.01)	0.999	(<0.001)	6.9	(0.2)	NG	
33	Rice	C246	V	7.8	(0.1)	0.02	(<0.01)	0.999	(<0.001)	9.8	(0.3)	0.045	(0.003)
4	Rice	ADRIA I21	VI	6.4	(<0.1)	6.4	(0.20)	0.960	(0.001)	6.0	(0.1)	NG	
Pre	Rice	ADRIA I21	VI	6.5	(0.1)	0.04	(<0.01)	0.999	(<0.001)	11.5	(0.1)	0.200	(0.019)

a Phylogenetic (panC) group.

b Average of three samples with standard deviation in brackets.

c Average within the time frame of experiment with standard deviation in brackets.

d NG: no observed growth within the time frame of experiment 28–49 days.

**Table 3:** Product characteristics, storage conditions and estimates of maximum specific growth rates (*μ_max_*) for challenge tests used to evaluate the performance of updated *Bacillus cereus sensu lato* growth models.

Exp. no.	Cooked food	Isolate	Group^a^	Measured characteristics^b^						Temp.^c^ (°C)		*μ_max_* (h^−1^)^b^	
				pH		% WPS		a_w_					
23	Bulgur	RIVM BC120	II	6.8	(<0.1)	5.0	(0.23)	0.967	(0.006)	11.7	(0.2)	0.084	(0.004)
9	Couscous	NVH 0861–00	II	5.4	(<0.1)	0.3	(<0.01)	0.992	(0.002)	11.2	(0.2)	0.072	(0.005)
Pre	Couscous	T101	IV	6.4	(<0.1)	1.8	(0.07)	0.990	(<0.001)	11.6	(0.2)	0.128	(0.010)
Pre	Couscous	C246	V	6.1	(<0.1)	6.4	(0.20)	0.952	(0.002)	11.2	(0.2)	NG^d^	
8	Couscous	C246	V	5.3	(<0.1)	8.0	(0.02)	0.944	(0.001)	6.6	(0.2)	NG	
7	Couscous	ADRIA I21	VI	6.4	(<0.1)	6.9	(0.3)	0.951	(0.001)	9.7	(0.5)	NG	
21	Mashed potato	T126	V	5.9	(<0.1)	2.2	(0.08)	0.987	(<0.001)	10.8	(0.6)	0.081	(0.001)
30	Mashed potato	C218	IV	5.9	(<0.1)	1.6	(0.03)	0.991	(<0.001)	10.8	(0.6)	NG	
Pre	Mashed potato	ADRIA I21	VI	6.2	(<0.1)	0.06	(<0.01)	0.999	(<0.001)	7.7	(0.1)	0.058	(0.008)
19	Pasta	ATCC 14579	IV	4.8	(<0.1)	4.6	(0.13)	0.971	(0.003)	11.0	(0.1)	NG	
5	Pasta	ADRIA I21	VI	6.6	(<0.1)	0.00	(<0.01)	0.999	(<0.001)	11.5	(0.1)	0.111	(0.031)
16	Pasta	KBAB4	VI	6.0	(<0.1)	6.2	(0.12)	0.959	(0.015)	10.8	(0.6)	NG	
29	Pasta	ADRIA I21	VI	5.0	(<0.1)	0.06	(>0.01)	0.996	(<0.001)	7.5	(0.4)	NG	
15	Pasta	KBAB4	VI	6.0	(<0.1)	4.7	(0.07)	0.970	(0.002)	6.7	(0.2)	0.011	(NA^e^)
2	Rice	NVH 0861–00	II	6.4	(<0.1)	3.4	(0.09)	0.978	(<0.001)	11.5	(0.1)	0.093	(0.006)
1	Rice	NVH 0861–00	II	7.8	(0.1)	0.02	(<0.01)	0.999	(<0.001)	9.8	(0.3)	0.080	(0.004)
Pre	Rice	NVH 0861–00	II	7.8	(0.1)	0.02	(<0.01)	0.999	(<0.001)	6.9	(0.2)	NG	
Pre	Rice	T101	IV	7.8	(0.1)	0.02	(<0.01)	0.999	(<0.001)	9.8	(0.3)	0.063	(0.006)
26	Rice	T126	V	6.6	(<0.1)	3.7	(0.08)	0.979	(0.001)	10.0	(0.5)	0.092	(0.012)
13	Rice	C246	V	7.8	(0.1)	0.02	(<0.01)	0.999	(<0.001)	6.9	(0.2)	NG	
3	Rice	ADRIA I21	VI	6.4	(<0.1)	4.8	(0.04)	0.971	(0.001)	6.0	(0.1)	NG	

a Phylogenetic (panC) group.

b Average of three samples with standard deviation in brackets.

c Average within the time frame of experiment with standard deviation in brackets.

d NG: no observed growth within the time frame of experiment 28 to 49 days.

e NA: not applicable as growth was only significant in one of the three samples.

Relevant ranges of temperature, pH and NaCl/a_w_ were determined from the seven preliminary challenge tests conducted with triplicate samples (Supplementary Table S1). Based on growth or no-growth results obtained in these preliminary challenge tests after storage for 2 weeks, three levels of temperature (7, 10, and 12°C) and pH (5, 6, and 7) and four levels of a_w_ (0.96, 0.97, 0.98, and 1.0) and four phylogenetic (*panC*) groups (II, IV, V, and VI) were selected. A screening design with temperature, pH and a_w_ as discrete numerical factors and phylogenetic (*panC*) groups as categorical factor was established using the DOE function in SAS JMP Pro (RRID:SCR_022199) (JMP^®^, Version 15. SAS Institute Inc., Cary, NC, 1989–2023). This constructing of a DOE resulted in 35 combinations (challenge tests) to be studied (Supplementary Table S2). The 35 challenge tests were then carried out using a broad range of cooked single component starchy foods, i.e., bulgur, couscous, mashed potatoes, pasta and rice, as substrate and by adjusting their pH and a_w_ to cover the values selected by the DOE. Bulgur (Polish, Coop, Coop Sweden), couscous (Polish, Coop, Coop Sweden), pasta (Italian soup horn, ØGO, Netto, Denmark) and rice (Basmati, Netto, Denmark) were purchased from local supermarkets and prepared in ion-exchanged water using the cooking instructions on the packages. Mashed potatoes were prepared by cutting peeled potatoes into small cubes and boiling for 20–30 min before mashing. When pH was adjusted, the cooking water was added either 2 M HCl or 2 M NaOH. Appropriate ratios between water and acid or water and base were determined in preliminary experiments for each commodity. When a_w_ was adjusted, NaCl (Supelco) was added to cooked starchy foods followed by thorough stirring. Appropriate NaCl quantities were determined from the expected moisture content of each commodity after cooking and from the desired a_w_–values. First, % water phase salt (WPS) was determined from the desired a_w_ –value using Equation (1) (Resnik and Chirife, 1988; Ross and Dalgaard, 2004) and then, % NaCl was calculated using Equation 2.


(1) $$\%WPS=8–140.7\cdot \left(a_{w}–0.95\right)–405.12\cdot {\left(a_{w}–0.95\right)}^{2}$$

(2) $$\%\mathrm{NaCl}=\frac{\%\mathrm{moisture}\cdot \%WPS}{100-\%WPS}$$

where WPS is water phase salt.

Approximately 900 g of product was prepared for each of the 42 challenge tests. Inoculation was done with individual *B. cereus s.l.* isolates from Table 1. The inocula were prepared by diluting spore stocks (see section 2.1.1) in saline and then heat-treating the spore suspension for 5 min at 80°C in a water bath primarily to simulate the cooking process used for the preparation of the sampled foods but also to inactivate vegetative cells. Products were added 9 times 1-mL aliquots of appropriately diluted inoculum with thorough mixing after each addition. This resulted in an initial concentration of approximately 10^2^ cfu/g. With this inoculation procedure, it was assumed that measured growth of *B. cereus s.l.* resulted from the inoculum as their concentration in the studied foods is low (Berthold-Pluta et al., 2019; Rahnama et al., 2022; Yu et al., 2020). This assumption was confirmed by selected uninoculated control samples where below 50 cfu/g of presumptive *B. cereus* were determined at the final storage time. Samples, each of 30 g, were then placed in sample bags (11,532,783, Fisherbrand, Fisher Scientific, Roskilde, Denmark). Following inoculation and packaging, products were stored aerobically at temperatures below 12°C as indicated in Tables 2, 3.

Applying the same procedure as above, five additional challenge tests were conducted with ready-to-cook foods bought in local supermarkets and consisting of composite foods with at least one starchy ingredient (yellow split pea stew, two potato/leek soups, curry soup, asparagus soup) (Table 4). All soups were stored in 50-mL centrifuge tubes (GR-227270, Greiner Bio-One, Kremsmünster, Austria) during the challenge test.

**Table 4:** Product characteristics, storage conditions and estimates of maximum specific growth rates (*μ_max_*) for *Bacillus cereus sensu lato* in challenge tests performed in the present study with composite foods containing at least one starchy ingredient.

Cooked food	Starch	Declared protein content (%)	Isolate(s)	Group^a^	Measured characteristics^b^						Temp.^c^ (°C)		*μ_max_*^b^ (h^−1^)	
					pH		WPS%		a_w_					
Yellow pea stew	Potato starch	3.0	KBAB4	VI	6.3	(<0.1)	1.1	(0.05)	0.994	(<0.001)	9.5	(0.1)	0.185	(0.013)
Meatballs	Wheat flour	14	RIVM BC120, C218, C246	II, IV, V	6.1	(<0.1)	2.5	(0.08)	0.986	(<0.001)	9.5	(0.1)	0.199	(0.027)
Liver pâté	Potato flour	9.5	RIVM BC120, C218, C246	II, IV, V	6.4	(<0.1)	2.6	(0.04)	0.978	(0.002)	6.0	(0.1)	0.063	(0.009)
Liver pâté	Potato flour	9.5	RIVM BC120, C218, C246	II, IV, V	6.3	(<0.1)	2.8	(0.06)	0.974	(0.003)	7.8	(0.3)	0.079	(0.002)
Potato/leek soup	Potato starch	0.7	KBAB4	VI	6.2	(<0.1)	0.9	(0.04)	0.995	(<0.001)	7.7	(0.3)	0.059	(0.001)
Potato/leek soup	Potato starch	0.7	C262	II	6.2	(<0.1)	0.9	(0.04)	0.995	(<0.001)	7.7	(0.3)	0.048	(0.003)
Curry soup	Mod. potato starch	1.5	NVH-0861-00	II	5.7	(<0.1)	1.2	(0.05)	0.991	(0.003)	7.8	(0.2)	NG^d^	
Asparagus soup	Wheat flour	0.7	C262	II	6.0	(<0.1)	0.8	(0.02)	0.993	(0.002)	7.8	(0.2)	NG	

a *Phylogenetic (panC) group*.

b *Average of three samples with standard deviation in brackets.*

c *Average within the time frame of experiment with standard deviation in brackets.*

d *NG: no observed growth within the time frame of experiments 29 days.*

Furthermore, challenge tests with ready-to-eat meat balls (*n* = 1) and ready-to-eat liver pâté (*n* = 2) were conducted using a slightly modified procedure because of their firmer texture (Table 4). These experiments were included to evaluate if composite foods rich in animal proteins or vegetable proteins from split peas resulted in faster growth than observed for single component starchy foods. For the challenge tests with meat balls and liver pâté, samples consisted of pieces of 15 ± 1 g placed in petri dishes (51,504, Fisherbrand) and inoculated with five droplets of 20 μL of a 1:1:1 cocktail of three isolates RIVM BC120 (group II), C218 (group IV), and C246 (group V).

For all the 50 challenge tests (Tables 2–4), sampling intervals were adjusted during storage time (2–7 weeks) based on sampling results and storage temperature. During each challenge test, samples were analyzed at six to 12 storage times. At each sampling point, three packages were picked for each tested product and randomly denoted A, B, and C and analyzed separately. With the exception of meat balls and liver pâté, where the entire 15-g samples were used, samples of 10 g of food were analyzed. All samples were diluted 10-fold in sterile physiological saline with peptone (PSP, 0.85% w/v with 0.1% Bacto Peptone, 211,677, Becton, Dickinson and Company, Sparks, United States) in blender bags with filter (02372, BagPage R, Interscience, Saint Nom la Bretêche, France) and homogenized for 30 s by using a Stomacher Lab Blender 400. Additional 10-fold dilutions of the homogenates were made in PSP. Viable counts of *B. cereus s.l.* were determined by spread plating suitable dilutions on Mannitol egg Yolk Polymyxin agar (MYP) (CM0929 + SR0047 + SR0099, Oxoid) or RAPID’B.cereus medium (12,007,305 + 12,007,306 + 12,007,307, Bio-Rad Laboratories, Copenhagen, Denmark) followed by enumeration of typical colonies after incubation at 30°C for 24 h.

#### 2.1.3 Storage temperatures and product characteristics in challenge tests

Data loggers (TinytagPlus, Gemini Data Loggers Ltd., Chichester, United Kingdom; Verdict 2 K: T, Verdict Systems BV, Aalten, The Netherlands) regularly recorded storage temperatures. The average temperature within the timeframe of each experiment was calculated and reported.

Product characteristics were determined by analysis of three uninoculated packages for each individual challenge test. The pH–value was measured with a HQ411D Laboratory Single Input instrument and a PHC724 probe (Hach Lange, Brønshøj, Denmark) using 5 g of product homogenized with 20 mL distilled water (NMKL 179, 2005). Salt was determined by automated potentiometric titration (785 DMP Titrino, Metrohm, Hesisau, Switzerland). Dry matter content was determined by oven drying at 105°C for 24 ± 2 h. The a_w_ –value was measured at 25°C applying the standard protocol for AQUALAB 4TE (Decagon devices Inc., Pullman, Washington, United States) after calibration of the instrument with distilled water and 40% potassium sulfate (1.05153.1000, Merck, Darmstadt, Germany).

#### 2.1.4 Fitting of growth curves

Growth kinetics of *B. cereus s.l.* were described by fitting the integrated and log_10_-transformed logistic model with delay, Equation 3 (Rosso et al., 1996) to log_10_-transformed cfu/g counts obtained as a function of storage time. Fitting was performed using non-linear regression with the method of least squares and the solver function in Microsoft Excel (RRID:SCR_016137). Fitted parameter values for initial cell concentration (Log *N_0_*, log_10_cfu/g), lag time (*t_lag_*, h), maximum specific growth rate (*μ_max_*, h^−1^) and maximum population density (Log *N_max_*, log_10_cfu/g) were determined for each growth curve collected from the samples denoted A, B, and C separately, resulting in three *μ*_max_–values for each challenge test. The *μ_max_*–values were reported as average and standard deviation of samples A, B, and C (Tables 2–4).


$$\mathrm{if}\;t<t_{\mathrm{lag}}\;\log\;\left(N_{t}\right)=\log\;\left(N_{0}\right)$$

(3) $$\begin{array}{l} \mathrm{if}\;t\geq t_{\mathrm{lag}}\;\log\;\left(N_{t}\right)=\\ \log\left(\frac{N_{\text{max}}}{1+\left(\left(\frac{N_{\text{max}}}{N_{0}}\right)-1\right)\cdot \exp\left(-\mu_{\text{max}}\cdot \left(t-t_{\mathrm{lag}}\right)\right)}\right) \end{array}$$

where *t* is the storage time (h) and *N_t_* is the cell concentration (cfu/g) at time *t*.

### 2.2 Evaluation of available growth models using growth responses from the present study

Ten predictive growth rate models, that included the effects of temperatures below 12°C, pH and a_w_ on the growth rate of either psychrotolerant or mesophilic-psychrotolerant intermediary thermotypes of *B. cereus s.l.*, were extracted from the scientific literature. One model was from ComBase: A Combined Database For Predictive Microbiology (RRID:SCR_008181), which are partly based on the work by Sutherland et al. (1996), while of the remaining models eight came from Carlin et al. (2013) and one from Zwietering et al. (1996) (Table 5). These models were used to predict responses for different *B. cereus s.l.* isolates based on product characteristics and storage temperature as described in Table 2.

**Table 5:** Evaluation of 10 existing *Bacillus cereus sensu lato* growth models using growth responses from the present study^a^.

Model	Group^b^	Isolate^c^	*μ_opt_* (h^−1^)	*T_min_* (°C)	*pH_min_*	*pH_max_*	*a_w min_*	*μ_max_* (h^−1^)			Growth/no-growth response			
								n	Bias factor (*B_f_*)	Accuracy factor (*A_f_*)	n	% correct	% fail-safe	% fail-dangerous
Carlin et al. (2013)	II	**RIVM BC120**	**2.61**	**1.4**	**4.68**	**9.80**	**0.946**	**12**	**2.12**	**2.12**	**21**	**62**	**38**	**0**
		**NVH 0861–00**	**2.72**	**5.1**	**4.62**	**9.80**	**0.950**	**12**	**0.96**	**1.42**	**21**	**62**	**38**	**0**
	IV	F4430/73	3.27	9.1	4.59	9.80	0.946	NA^d^	NA	NA	NA	NA	NA	NA
		ATCC 14579	2.76	7.8	4.60	9.80	0.956	NA	NA	NA	NA	NA	NA	NA
	V	**F2769/77**	**2.81**	**5.1**	**4.87**	**9.80**	**0.956**	**12**	**0.75**	**1.66**	**21**	**67**	**33**	**0**
		**NVH 141**	**2.82**	**5.2**	**4.69**	**9.80**	**0.949**	**12**	**0.87**	**1.44**	**21**	**62**	**38**	**0**
	VI	KBAB4	1.83	3.9	4.85	9.80	0.964	NA	NA	NA	NA	NA	NA	NA
		ADRIA I21	2.29	3.3	4.96	9.80	0.973	NA	NA	NA	NA	NA	NA	NA
ComBase (2024)	NR^e^	NR	NR	5.0^f^	4.90^f^	7.40^g^	0.94^f^	11	1.41	1.47	17	65	35	0
Zwietering et al. (1996)	NR	**NCM** ^**h**^	**2.00**	**0.0**	**4.90**	**NI** ^**i**^	**0.950**	**12**	**1.33**	**1.41**	**21**	**67**	**33**	**0**

a Average of data in Table 2 were used.

b Phylogenetic (panC) group.

c Models displayed in bold were selected for further evaluation.

d NA: evaluation not applicable, as growth was observed below T_min_ or a_w min_ of the model.

e NR: not reported.

f Minimum levels that can be used for prediction.

g Maximum level that can be used for prediction.

h NCM: model developed for naturally contaminated milk.

i NI: not included in model.

Carlin et al. (2013) developed cardinal parameter models including the effect of temperature (*T*), pH and a_w_ (Equations 4 and 5) on *μ_max_*–values of *B. cereus s.l.* isolates from different phylogenetic *panC* groups. The eight models included in the present study (Table 5) had *T_min_*–values from 1.4 to 9.1°C, *pH_min_*–values from 4.59 to 4.96 and *a_w min_*–values from 0.946 to 0.973 (Carlin et al., 2013).


(4) $$\mu_{\text{max}}=\mu_{\mathrm{opt}}\cdot CM_{2}\;\left(T\right)\cdot CM_{1}\left(pH\right)\cdot CM_{1}\left(a_{w}\right)$$

(5) $$\begin{array}{l} CM_{n}\left(X\right)=\left\{\begin{array}{ccc} 0 & & ;\\ & \frac{\left(X-X_{\text{max}}\right)\cdot {\left(X-X_{\text{min}}\right)}^{n}}{{\left(X_{\mathrm{opt}}-X_{\text{min}}\right)}^{n-1}\cdot \left[\left(X_{\mathrm{opt}}-X_{\text{min}}\right)\cdot \left(X-X_{\mathrm{opt}}\right)-\left(X_{\mathrm{opt}}-X_{\text{max}}\right)\cdot \left(X_{\mathrm{opt}}+X_{\text{min}}-nX\right)\right]} & ;\\ 0 & & ; \end{array}\right.\begin{array}{ccc} & X\leq X_{\text{min}} & \\ & X_{\text{min}}<X<X_{\text{max}} & \\ & \geq X_{\text{max}} & \end{array} \end{array}$$

For growth of naturally occurring *B. cereus s.l.* in milk, Zwietering et al. (1996) suggested a cardinal parameter model like Equation 4 and with terms for temperature, pH and a_w_ that were simpler than indicated by Equation 5. This model used *T_min_* = 0.0°C; *pH_min_* = 4.9 and *a_w min_* = 0.95.

These 10 growth rate models were evaluated by using 21 growth/no-growth responses and corresponding product characteristics as determined in the present study (see Section 2.1 and Table 2). This screening of growth rate models was used to exclude the models with poor, or no potential of improved performance by product calibration and/or expansion with terms for interactions between *T*, *pH* and *a_w_*.

### 2.3 Indices used to evaluate the performance of growth and growth boundary models

The performance of growth rate models was evaluated by comparison of observed and predicted *μ_max_*–values. Bias factor (*B_f_*; Equation 6) and accuracy factor (*A_f_*; Equation 7) values were calculated and compared with limits previously used for evaluating growth rate models for various bacteria: 0.95 < *B_f_* < 1.11 indicate a good model performance, with *B_f_* in the intervals of 1.11–1.43 or 0.87–0.95 corresponding to acceptable model performance and *B_f_* < 0.87 or > 1.43 considered as unacceptable model performance (Mejlholm et al., 2010).


(6) $$B_{f}\left(\mu_{\text{max}}\right)=10^{\left(\sum \log\left(\mu_{\max\mathrm{predicted}}/\mu_{\max\mathrm{observed}}\right)/n\right)}$$

(7) $$A_{f}\left(\mu_{\text{max}}\right)=10^{\left(\sum |\log\left(\mu_{\max\mathrm{predicted}}/\mu_{\max\mathrm{observed}}\right)|/n\right)}$$

In addition, *A_f_* > 1.5 indicate poor model precision or a systematic deviation between observed and predicted *μ_max_*–values (Mejlholm and Dalgaard, 2013). Predicted and observed growth and no-growth responses were evaluated by calculating the percentage of samples that were correctly predicted. Incorrect predictions were considered as fail-safe (growth predicted when no-growth was observed) or fail-dangerous (no growth predicted when growth was observed). Criteria corresponding to good, acceptable and unacceptable model performance have not been established for the percentage of correct, fail-safe and fail-dangerous predictions. Nevertheless, when evaluating different models and using the same data set these indices allow the performance of models to be ranked. Larger validation studies found the better models to have >75% correct, < 15% fail-safe and < 10% fail-dangerous predictions (Koukou et al., 2022; Martinez-Rios et al., 2020; Mejlholm et al., 2010). Ideally, models should provide 100% correct, 0% fail-safe and 0% fail-dangerous predictions but when product characteristics are close to the growth boundary a small percentage of fail-safe and fail-dangerous predictions can be observed, even for precise models, due to for example variability in product characteristics. Therefore, it is particularly important to indicate if fail-dangerous predictions are close to the growth boundary and this can be done by using the *ψ*–value (see section 2.4, Equation 12) which has a value of 1.0 at the growth boundary (Mejlholm and Dalgaard, 2009).

### 2.4 Evaluation of models updated by product calibration and expansion with terms for interaction between environmental factors

Even though *B_f_* –value, *A_f_* –value and proportion of correct, fail-safe and fail-dangerous predictions of growth/no-growth responses are normally used as model performance indices, they were calculated here to be able to select the most promising models. Different approaches for model improvement were applied. For each of the selected literature models, 82 new models were developed in the following way. One model was developed by product calibrating the *μ_opt_*–value using Equation 8 to create *μ_opt-C_*. By keeping the *μ_opt_*–value unchanged and expanding the model with the interaction term ξ (Equation 9) using three different values of *n* in Equation 11, for the effect of interaction between temperature, pH and a_w_, resulted in 3^3^ = 27 different models. Another 2 × 27 = 54 models were developed, 27 by first product calibrating *μ_opt_* and then expanding with the interaction term ξ and 27 by first expanding with the interaction term ξ and then product calibrating *μ_opt_*.

Product calibration of the selected Carlin et al. (2013) and Zwietering et al. (1996) models was performed by dividing the original *μ_opt_* –values for each model with the *B_f_* –value determined for the specific model (Equation 8).


(8) $$\mu_{\mathrm{opt}-C}=\frac{\mu_{\mathrm{opt}}}{B_{f}}$$

where *μ_opt-C_* is the maximum specific growth rate (h^−1^) after product calibration and at the optimum growth temperature as suggested by Koukou et al. (2021).

*ξ* in Equation 9 described the effect of interactions between the environmental factors and its effect was modeled as previously reported by using the Le Marc approach (Le Marc et al., 2002). The value of *ξ* was between 0 and 1 and calculated according to Equations 10–12.


(9) $$\mu_{\text{max}}=\mu_{\mathrm{opt}}\cdot CM_{2}\;\left(T\right)\cdot CM_{1}\left(pH\right)\cdot CM_{1}\left(a_{w}\right)\cdot \xi$$

(10) $$\xi (\varphi \left(T,pH,a_{w}\right)=\left\{\begin{array}{ccc} 1 & ; & \psi \leq 0.5\\ 2\left(1-\psi \right) & ; & 0.5\leq \psi \leq 1\\ 0 & ; & \psi \geq 1 \end{array}\right.$$

where *ξ*(*ϕ*(T, pH, a_w_) is the term describing the effects of interactions between environmental factors on *μ_max_*. For temperature, pH and a_w_ the contribution of each of these terms in Equation 9 to the interaction term (ξ, Equation 10) was calculated by using Equation 11 and Equation 12. Le Marc et al. (2002) applying a value of 2.0 for *n* in Equation 11, however, in the present study the effect of using values of 1, 2 or 3 was evaluated as described below.


(11) $$\varphi_{\mathrm{Environmental}\;\mathrm{term}}={\left(1-\sqrt{\mathrm{Environmental}\;\mathrm{term}}\right)}^{n}$$

(12) $$\psi =\underset{i}{\sum }\frac{\varphi_{e_{i}}}{2\underset{j\neq i}{\prod }\left(1-\varphi_{e_{i}}\right)}$$

where *e_i_* represents the environmental factors and *ϕ_e_* the contribution of each environmental term to the effect of interactions between the factors.

The *ψ*–value provides a measure of how far a specific set of environmental factors is from the growth boundary (Mejlholm and Dalgaard, 2009) and a *ψ*–value higher than 1.0 indicated no growth (Equation 10).

To find the most promising interaction terms for the effect of *T*, *pH* and *a_w_* in the better performing models, all the 27 combinations, which resulted from using values of 1, 2, or 3 for *n* in Equation 11 when used for *T*, *pH* or *a_w_*, respectively, were tested. For each model, the *B_f_* – and *A_f_* –values as well as the percentages of correct, fail-safe and fail-dangerous predictions of growth/no-growth responses were calculated using the dataset in Table 2. This approach was performed both before and after product calibration of the models. Models with good or acceptable *B_f_* – and *A_f_* –values (see section 2.3), ≥ 75% correct and ≤ 5% fail-dangerous predictions for growth/no-growth responses were selected for further evaluation.

### 2.5 Evaluation of the most promising of the updated models with independent data

All new models, constructed as described in Section 2.4 and fulfilling the acceptability criteria for performance, were selected as promising models. Performance of these promising models were evaluated using the independent data reported in Table 3. *B_f_* – and *A_f_* –values (Equations 6 and 7) and proportion of correct, fail-safe and fail-dangerous predictions of growth/no-growth responses were calculated and used as model performance indices with the purpose of selecting two of the models for further evaluation using growth/no-growth responses reported in the scientific literature.

### 2.6 Growth responses and product characteristics extracted from available studies

A total of 33 kinetic responses, for more than 12 different isolates of *B. cereus s.l.*, in a range of single component and composite starchy foods were extracted from five available studies and 10 ComBase records. The studies with single component starchy foods included mashed potatoes from powder, cooked rice, cooked noodles, sliced bread and potato purée whereas the composite starchy foods included meat loaf, composite fried rice meal, pizza, meat lasagna, cottage pie and vegetable pie (Table 6). Exclusively, responses reported for storage temperatures of max 12°C were studied and exclusively for psychrotolerant or mesophilic-psychrotolerant intermediary thermotypes, i.e., strains belonging to the phylogenetic (*panC*) groups II, IV, V, and VI. When phylogenetic groups were not reported, then the thermo-type of strains was considered psychrotolerant when growth was observed below 10°C or mesophilic-psychrotolerant intermediary when growth was observed at 10°C but not below (Table 6). Product characteristics (pH, NaCl/a_w_) and storage temperature were recorded for kinetic responses extracted from literature (Table 6). When no information regarding NaCl/a_w_ or pH was provided, then an average value was assumed from reported values for a similar type of food. Other environmental factors, including organic acids, were not mentioned for any of the eight studies analyzed and, therefore, assumed not to be present. When a_w_ was not reported, it was estimated using concentrations of NaCl and moisture to determine % WPS (Equation 13) and converting this to a_w_ using Equation 14 (Resnik and Chirife, 1988; Ross and Dalgaard, 2004) shown below.


(13) $$\%WPS=\frac{100\cdot \%\mathrm{NaCl}}{\%\mathrm{moisture}+\%\mathrm{NaCl}}$$

(14) $$a_{w}=1–0.0052471\cdot \%WPS–0.00012206\cdot \%{WPS}^{2}$$

**Table 6:** Growth responses (*μ_max_*, of *Bacillus cereus* *sensu lato* and product characteristics of starchy foods extracted from available studies.

Reference	Strain(s)	Thermotype^a^ (phylogenetic *panC* group)	Food^b^	T (°C)	Added salt (% w/w)	Water phase salt (%)	a_w_^c^	pH^c^	*μ_max_* (h^−1^)	
Mahakarnchanakul and Beuchat (1999)	F3802A/84	Psychrotolerant (?)	Mashed potatoes from powder (S)	10	0	NR^d^	**0.999**	5.8	0.082	A^e^
			Mashed potatoes from powder (S)	10	2	NR	**0.987**	5.8	0.045	A
			Mashed potatoes from powder (S)	10	4	NR	**0.975**	5.8	NG^f^	A
	B4ac-1	Intermediary (IV)	Mashed potatoes from powder (S)	10	0	NR	**0.999**	5.8	0.023	A
			Mashed potatoes from powder (S)	10	2	NR	**0.987**	5.8	NG	A
			Mashed potatoes from powder (S)	10	4	NR	**0.975**	5.8	NG	A
Ultee et al. (2000)	IFR-NL 94–25	Psychrotolerant (?)	Cooked rice (S)	8	0	NR	**0.999**	**6.5**	0.046	B
			Cooked rice (S)	8	0	NR	**0.999**	**6.5**	0.055	B
Thorsen et al. (2009)	Strain 37 (*B. weihenstephanensis*)	Psychrotolerant (VI)	Meat loaf^g^ (C)	8	1.2	2.1	0.988	6.2	0.030	B
			Meat loaf^g^ (C)	8	1.2	2.1	0.988	6.2	0.029	B
	MC118 (*B. weihenstephanensis*)	Psychrotolerant (VI)	Meat loaf^g^ (C)	8	1.2	2.1	0.988	6.2	0.022	B
			Meat loaf^g^ (C)	8	1.2	2.1	0.988	6.2	0.028	B
	INRA 161 (*B. weihenstephanensis*)	Psychrotolerant (VI)	Meat loaf^g^ (C)	8	1.2	2.1	0.988	6.2	0.020	B
			Meat loaf^g^ (C)	8	1.2	2.1	0.988	6.2	0.031	B
Kang et al. (2010)	ATCC 11778	Intermediary (IV)	Cooked noodles (S)	8	NR	0	1.00	7.4	NG	C
			Cooked noodles (S)	10	NR	0	1.00	7.4	NG	C
			Cooked rice (S)	8	NR	0	0.99	6.6	NG	C
			Cooked rice (S)	10	NR	0	0.99	6.6	NG	C
			Sliced bread (S)	8	NR	0.17	0.95	7.7	NG	C
			Sliced bread (S)	10	NR	0.17	0.95	7.7	NG	C
Tirloni et al. (2019)	GPe2	Intermediary (?)	Composite fried rice meal (C)	10	NR	NR	0.972	6.7	0.041	B
	ATCC 14579 (Type strain *B. cereus*)	Intermediary (IV)	Composite fried rice meal (C)	10	NR	NR	0.972	6.7	0.054	B
	R1	Intermediary (?)	Composite fried rice meal (C)	10	NR	NR	0.972	6.7	0.111	B
ComBase (2023a)	FMBRA strains 432, 433, 434, 436	Not known	Pizza (ID: O281_5) (C)	10	NR	NR	0.994	5.1	0.023	D
ComBase (2023b)	FMBRA strains 432, 433, 434, 436	Not known	Pizza (ID: O281_6) (C)	10	NR	NR	0.983	5.1	0.007	D
ComBase (2023c)	Not specified (*B. cereus*)	Intermediary (?)	Meat lasagne (ID: P176_3) (C)	8	NR	1.8	0.990	5.8	NG	D
ComBase (2023d)	Not specified (*B. cereus*)	Intermediary (?)	Meat lasagne (ID: P176_2) (C)	12	NR	1.8	0.990	5.8	0.115	D
ComBase (2023e)	Not specified (*B. cereus*)	Intermediary (?)	Cottage pie (ID: P175_3) (C)	8	NR	NR	**0.997**	5.9	NG	D
ComBase (2023f)	Not specified (*B. cereus*)	Intermediary (?)	Cottage pie (ID: P175_2) (C)	12	NR	NR	**0.997**	5.9	0.155	D
ComBase (2023g)	Not specified (*B. cereus*)	Intermediary (?)	Vegetable pie (ID: P178_3) (C)	8	NR	1.8	0.990	5.6	NG	D
ComBase (2023h)	Not specified (*B. cereus*)	Intermediary (?)	Vegetable pie (ID: P178_2) (C)	12	NR	1.8	0.990	5.6	0.073	D
ComBase (2023i)	INRAAV-Z4222 (*B. cereus*)	Psychrotolerant (?)	Potato purée (ID: Car_69) (S)	7	NR	NR	**0.997**	5.8	0.042	D
ComBase (2023j)	INRAAV-Z4222 (*B. cereus*)	Psychrotolerant (?)	Potato purée (ID: Car_68) (S)	11	NR	NR	**0.997**	5.8	0.131	D

a As indicated in the cited references or evaluated in the present study as psychrotolerant when growth was observed below 10°C or intermediary when growth was observed at 10°C but not below. The (?) indicate that information of *panC* group was not available.

b (S): assumed single component starchy food and (C): assumed composite starchy food.

c Bold types: assumed values. See explanation in section 2.1.

d NR: not reported.

e Capital letters indicate how *μ_max_* was estimated in the present study. A: counts read from table were fitted, B: counts read from figure were fitted, C: generation time or no growth reported in cited reference, D: counts reported in the ComBase browser were fitted.

f NG: no growth.

g Added 7% bread crumbs and 2% wheat flour. Packaged in 2% O_2_ and 20% CO_2_.

where WPS is water phase salt.

### 2.7 Evaluation of the two best performing of the updated models with data from the scientific literature and for composite starchy foods

Evaluation of the two most promising models were performed using the scientific literature data shown in Table 6 and the composite starchy foods from the present study reported in Table 4. For these evaluations, *B_f_* – and *A_f_* –values (Equations 6 and 7) and proportion of correct, fail-safe and fail-dangerous predictions of growth/no-growth responses were calculated and used as model performance indices with the purpose of selecting the best model to use for predicting safe shelf-lives.

## 3 Results and discussion

### 3.1 Screening of existing growth models using the growth responses in Table 2

The initial 21 challenge tests, which included nine *B. cereus s.l.* isolates, five starchy food products, storage temperatures from 6.6 to 11.7°C, pH 4.8–7.8 and % WPS of 0.02–9.0 with a_w_–values of 0.935–0.999, resulted in nine tests with no-growth responses and 12 tests with average *μ_max_* –values of 0.016–0.200 h^−1^ (Table 2). When compared to the experimental data, two models were excluded from further evaluation due to observed growth at temperatures ≤7.7°C (Table 2), which is lower than the *T_min_*–value of strains F4430/73 (*T_min_* = 9.1°C) and ATCC 14579 (*T_min_* = 7.8°C) (Carlin et al., 2013). Another two of the 10 studied models from the literature were excluded from further evaluation due to observed growth in bulgur adjusted to a_w_ of 0.957 and in pasta adjusted to a_w_ of 0.962 (Table 2), which were below the *a_w min_* –values of 0.964 and 0.973 for the strains KBAB4 and ADRIA I21, respectively (Carlin et al., 2013). The model from ComBase was also excluded from further study. This model had a proportion of correct growth/no-growth predictions of 65% (Table 5) being lower than 75% which is aimed for in validation studies. Moreover, as this model is not a cardinal parameter-type model, and its model parameter values are not known (ComBase, 2024), it was not possible to expand the model with an interaction term to improve the percentage of correct and fail-safe predictions.

The five remaining models were the models for group II strains RIVM BC120 and NVH 0862–00, group V strains F2769/77 and NVH 141 (Carlin et al., 2013) and the model in Zwietering et al. (1996). They are all cardinal parameter models and had no fail-dangerous predictions of the growth/no-growth responses (Table 5). Two of these models, i.e., the Carlin et al. (2013) model for group II strain RIVM BC120 and the model in Zwietering et al. (1996), both had *B_f_* – and *A_f_* –values that were well above 1.0 and close to each other (Table 5). This does not necessarily disqualify these models from further studies as such a situation has previously been solved by product calibration of the model where the *μ_opt_*–value is calibrated to include the effect of specific foods (Dalgaard and Mejlholm, 2019; Koukou et al., 2021; Rosso et al., 1996). High *B_f_* – and/or *A_f_* –values have also been linked to evaluation of predictive growth rate models without an interaction term (Mejlholm and Dalgaard, 2009). When approaching the growth boundary, growth rates are often reduced due to the interaction between environmental factors and if not accounted for in a predictive model, then growth rates can be over-predicted resulting in increased *B_f_* – and *A_f_* –values and a high proportion of fail-safe predictions of the growth/no-growth responses (Mejlholm and Dalgaard, 2009). Neither the Carlin et al. (2013) models nor the Zwietering et al. (1996) model included an interaction term, suggesting expansion of these models with this term could be an option to decrease the number of fail-safe and increase the number of correct predictions of the growth/no-growth responses. Importantly, Carlin et al. (2013) and Zwietering et al. (1996) models were developed in markedly different ways. Carlin et al. (2013) developed models for growth rates of individual isolates in BHI broth whereas Zwietering et al. (1996) estimated cardinal parameter values from data for growth of naturally occurring *B. cereus* in milk.

### 3.2 Product calibration and expansion of models with interaction term using growth responses

Of the overall 410 models (82 modifications of each of the five original models) that were tested using the growth responses in Table 2, nine models complied with the criteria of having a good or an acceptable *B_f_* –value (0.87 ≤ *B_f_* ≤ 1.43), an acceptable *A_f_* –value (*A_f_* ≤ 1.5) and at the same time resulting in ≤5% fail-dangerous and ≥ 75% correct predictions of growth/no-growth responses (Table 7). All nine of the best performing models were derived from the two original models with the lowest *T_min_*–values, i.e., 1.4°C for the group II strain RIVM BC120 model (Carlin et al., 2013) and 0.0°C for the model in Zwietering et al. (1996) (Table 5). As growth responses at low temperatures was predicted this is probably to be expected. Importantly, all these nine models included a term for the growth inhibiting effect of interactions between the environmental factors, temperatures, pH and a_w_ (Table 7). This confirmed the importance of taking the effect of interactions into account when growth responses are predicted as previously observed, e.g., for mesophilic *B. cereus* (Le Marc et al., 2021), *L. monocytogenes* (Augustin and Carlier, 2000; Le Marc et al., 2002; Mejlholm and Dalgaard, 2009) and non-proteolytic *Clostridium botulinum* (Koukou et al., 2021).

**Table 7:** Characteristics of the nine best performing and updated *Bacillus cereus sensu lato* growth models^a^.

Model	*PanC* group^b^	Isolate	Approach for model development	*μ_opt_/μ_opt-C_* (h^−1^)	Interaction term			*μ_max_* (h^−1^)			Growth/no-growth (n = 21)		
					n(T)	n(pH)	n(aw)	n	Bias factor (*B_f_*)	Accuracy factor (*A_f_*)	% correct	% fail-safe	% fail-dangerous (*Ψ*)
Carlin et al. (2013)	II	RIVM BC120	Calibration+Interaction	1.23	3	1	3	12	0.87	1.47	86	14	0
			Calibration+Interaction	1.23	3	2	3	12	0.90	1.45	81	19	0
			Calibration+Interaction	1.23	3	3	3	12	0.90	1.45	76	24	0
			Interaction+Calibration	1.41	3	1	3	12	1.00	1.39	86	14	0
			Interaction+Calibration	1.35	3	2	3	12	1.00	1.41	81	19	0
			Interaction+Calibration	1.35	3	3	3	12	1.00	1.41	76	24	0
Zwietering et al. (1996)	NR^c^	NCM^d^	Interaction	2.00	3	3	3	11	1.23	1.55	76	19	5 (1.03)
			Calibration+Interaction	1.52	3	3	3	11	0.93	1.53	76	19	5 (1.03)
			Interaction+Calibration	1.63	3	3	3	11	1.00	1.50	76	19	5 (1.03)

a Models were updated by interaction terms and product calibration of *μ_opt_*. Data in Table 2 were used to study the updated models.

b Phylogenetic group.

c NR: not reported.

d NCM: model developed for naturally contaminated milk.

The *μ_opt_*–value was calibrated for eight of the nine best performing models (Table 7) indicating that growth rates of *B. cereus s.l.* in starchy foods differ from growth rates in the BHI broth or milk used for development of the original models (Carlin et al., 2013; Zwietering et al., 1996). Only, one of nine best performing models included the original *μ_opt_*–value from milk (Table 7; Zwietering et al., 1996).

As changing the *μ_opt_* –value does not affect the cardinal parameter values, which define the growth/no-growth conditions, product calibration as the sole approach for updating models (i.e., approach i, see section 2.4) could not improve the predictions of the growth/no-growth responses. However, product calibration in combination with expanding the original models with an interaction term (i.e., approaches iii and iv, see section 2.4) was very effective, as resulting in acceptable performance of eight of nine models in Table 7 which were updated in this way. Half of these eight models were a result of first calibrating the *μ_opt_* –value of the original models and then expanding the model with an interaction term, while the other four models were expanded with an interaction term before calibration of *μ_opt_* –values (Table 7). Hence, no clear picture on best practice could be deduced indicating that it could be depending on the specific model.

The six models, originating from Carlin et al. (2013) group II strain RIVM BC120 model, kept 0% fail-dangerous predictions after updating, suggesting the best model for the purpose of predicting the growth/no-growth response of *B. cereus s.l.* in starchy foods would be found among these six. However, the 5% fail-dangerous predictions of growth/no-growth responses obtained for the updated Zwietering et al. (1996) models, do not necessarily disqualify these models from further studies. The result of 5% fail-dangerous predictions of no-growth, when growth was observed, corresponded to one of the challenge tests, i.e., Exp. no. 20 (Table 2). In this challenge, with cooked bulgur inoculated with group VI strain ADRIA I21, the *ψ*–value was determined to be 1.03 (Table 7) with the updated models when the average measurements of storage temperature of 11.7°C, pH of 6.8 and a_w_ of 0.958 (Table 2) were applied. As a value of 1.0 indicates the growth boundary (Le Marc et al., 2024; Mejlholm and Dalgaard, 2009), the value 1.03 predicted no-growth. As the a_w_ –value of this challenge is close to the *a_w min_* –value of 0.950 for the model even small deviations in the a_w_ measurement can change the *ψ*–value from predicting no-growth to predicting growth, e.g., using the highest a_w_ –value of 0.959, measured in this case, would have resulted in a *ψ*–value of 0.96, thereby, predicting a growth response in this sample. Therefore, none of the three models originating from the Zwietering et al. (1996) were disqualified and all nine updated models were studied further as promising candidates being evaluated using the independent experimental data shown in Table 3.

### 3.3 Evaluation of the most promising of the updated models using growth responses in Table 3

The 21 performed challenge tests, used as the independent growth/no-growth responses (Table 3) in the evaluation, included the same nine *B. cereus s.l.* isolates and the same five starchy food products as used for the updating of literature models. Regarding storage temperature, pH, % WPS and measured a_w_, all levels were within the ranges used for updating the models (Tables 2, 3). Challenge tests resulted in ten no-growth responses and 11 growth responses with average *μ_max_*–values of 0.011–0.128 h^−1^ (Table 3).

**Figure 1:**
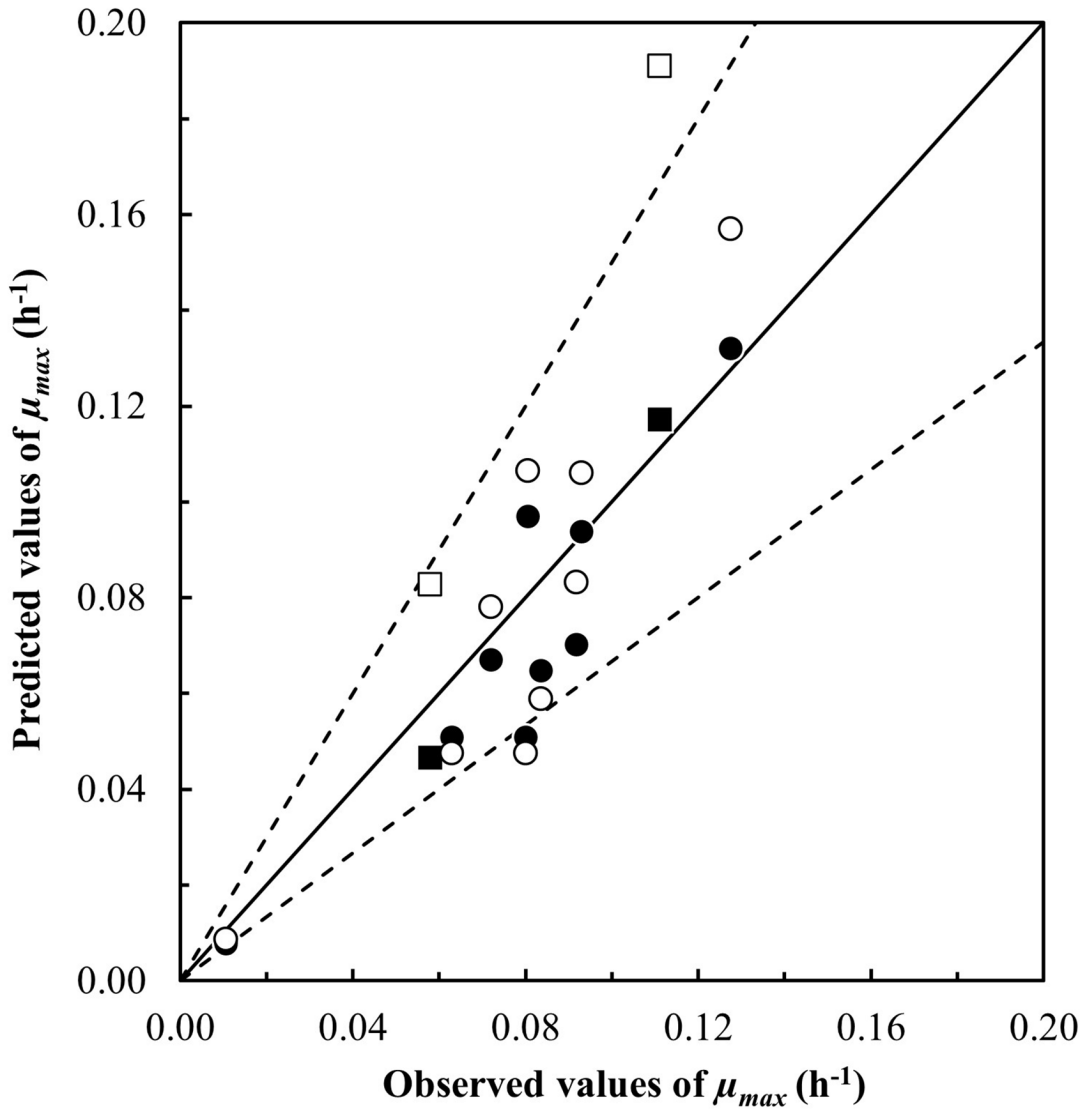
Comparison of observed and predicted maximum specific growth rates (*μ_max_*, h^−1^) for *Bacillus cereus sensu lato* in starchy foods (Table 3). Growth was predicted using (i) the updated Carlin et al. (2013) model for the *panC* group II strain RIVM BC120 (●, ■) with *B_f_*– and *A_f_* –values of 0.87 and 1.21 and (ii) the updated model from Zwietering et al. (1996) (○, □) with *B_f_* – and *A_f_* –values of 1.01 and 1.32. Square symbols represent samples for the *panC* group VI strain ADRIA I21. The solid line represents the line of perfect match and the dotted lines represent ± *A_f_* –value of 1.5.

Four of the nine studied models performed with *B_f_*–values between 0.87 and 1.01 as well as *A_f_*–values between 1.15 and 1.32 indicating acceptable to good performance for prediction of *μ_max_*–values (h^−1^) (models in bold, Table 8). Of these four models, two stood out with better results for the prediction of the growth/no-growth responses and resulted in more than 75% correct, less than 25% fail-safe and no fail-dangerous predictions (models with *, Table 8). This was an improvement of the number of correct predictions of 14 percentage points for both the Carlin et al. (2013) model for group II strain RIVM BC120 as well as for the model in Zwietering et al. (1996) (Tables 5, 8). Figure 1 compares observed and predicted *μ_max_*–values for these two models and illustrates, that predictions obtained using the updated Zwietering et al. (1996) model, on average were less biased with equal number of data points scattered around the line of perfect match, though with two results positioned further above the line as indicated with square symbols in Figure 1 (□). These two growth responses were both from challenge tests with the *panC* group VI strain ADRIA I21 in samples with no added NaCl (a_w_, 0.999) having pH–values (6.2 and 6.6) around the optimal of 6.4 for this strain (Carlin et al., 2013) and stored at 7.7 and 11.5°C, respectively (Table 3). However, as shown in Figure 1 (■), these two growth responses did not deviate markedly from the predicted *μ_max_*–value when using the updated Carlin et al. (2013) model for *panC* group II strain RIVM BC120. The difference appeared to be related to the environmental term for a_w_, CM_1_(a_w_), where the predicted values for the updated Carlin et al. (2013) model for *panC* group II strain RIVM BC120 were lower compared to values obtained using the updated Zwietering et al. (1996) model (results not shown). The cardinal parameter *a_w opt_* has the value of 1.0 in the Zwietering et al. (1996) model meaning that *a_w max_* becomes irrelevant. With *a_w opt_* and *a_w max_* of 0.997 and 1.0, respectively, in the Carlin et al. (2013) model for *panC* group II strain RIVM BC120, the consequence is that a_w_–values of more than 0.9985 will result in a shift toward lower CM_1_(a_w_) and lower predicted *μ_max_*–values for this model compared to the Zwietering et al. (1996) model.

**Table 8:** Evaluation of the nine most promising of the updated *Bacillus cereus sensu lato* growth models using growth responses (*μ_max_*) generated in this study^a^.

Model	Group^b^	Isolate	Approach for model development^c^	*μ_opt_* (h^−1^)	Interaction term			Evaluation					
								*μ_max_* (h^−1^)			Growth/no-growth (n = 21)		
					n(T)	n(pH)	n(a_w_)	n	Bias factor (*B_f_*)	Accuracy factor (*A_f_*)	% correct	% fail-safe	% fail-dangerous
Carlin et al. (2013)	II	RIVM BC120	Calibration+Interaction	1.23	3	1	3	11	0.76	1.33	76	24	0
			Calibration+Interaction	1.23	3	2	3	11	0.85	1.21	71	29	0
			Calibration+Interaction	1.23	3	3	3	11	0.85	1.21	67	33	0
			Interaction + Calibration*	**1.41**	**3**	**1**	**3**	**11**	**0.87**	**1.21**	**76**	**24**	**0**
			**Interaction + Calibration**	**1.35**	**3**	**2**	**3**	**11**	**0.93**	**1.15**	**71**	**29**	**0**
			**Interaction + Calibration**	**1.35**	**3**	**3**	**3**	**11**	**0.93**	**1.15**	**67**	**33**	**0**
Zwietering et al. (1996)	NR^d^	NCM^e^	Interaction*	**2.00**	**3**	**3**	**3**	**11**	**1.01**	**1.32**	**81**	**19**	**0**
			Calibration+Interaction	1.52	3	3	3	11	0.77	1.39	81	19	0
			Interaction+Calibration	1.63	3	3	3	11	0.83	1.34	81	19	0

a Data in Table 3 were used.

b Phylogenetic group.

c Models displayed in bold had acceptable *B_f_* – and *A_f_* –values. Models with * had both acceptable *B_f_* – and *A_f_* –values as well as acceptable predictions of growth/no growth responses.

d NR: not reported.

e NCM: model developed for naturally contaminated milk.

Nevertheless, both models performed within an acceptable range for starchy foods, and both were additionally evaluated using growth responses partly reported in the scientific literature (Table 6) (*n* = 33), partly generated in the present study (Table 4) (*n* = 8).

### 3.4 Evaluation of the two most promising updated models using growth responses from the literature (Table 6) and challenge tests in composite foods (Table 4)

The dataset extracted from the scientific literature consisted of 33 growth/no-growth responses, leading to the acquisition of 21 *μ_max_* –values and 12 no-growth responses to be included in the evaluation (Table 6). Different sub-datasets of growth/no-growth responses were created for the evaluation based on data in Table 6; one for each of the two thermotypes psychrotolerant (*n* = 13) and intermediary (*n* = 18), one for single component starchy foods (*n* = 16), one for composite starchy foods (*n* = 17), one for meat loaf (*n* = 6) and a final sub-dataset excluding the data from meat loaf (*n* = 27) (Table 9). Using the 21 *μ_max_*–values, the updated model from Carlin et al. (2013) for *panC* group II strain RIVM BC120 performed better than the updated Zwietering et al. (1996) model with *B_f_* – and *A_f_* –values closer to the acceptance criteria (Table 9). This applied regardless of sub-dataset suggesting a systematic difference resulting in generally higher *μ_max_* predictions for the updated Zwietering et al. (1996) model. When looking closer into this difference, it revealed that the predicted *ψ*–values were lower for 27 out of the 33 challenge tests when applying the updated Zwietering et al. (1996) model (data not shown) indicating a lower dampening effect of the interaction term compared to the updated Carlin et al. (2013) model for *panC* group II strain RIVM BC120. On the other hand, the higher dampening effect, seen for the updated Carlin et al. (2013) model for *panC* group II strain RIVM BC120, resulted in two predictions of fail-dangerous growth responses (Table 9). When looking closer into these two fail-dangerous predictions, both cases appeared to concern pizzas, one with pH 5.1 having a_w_ –value of 0.983 (ComBase, 2023b) and another with pH 5.1 and a_w_–value of 0.994 (ComBase, 2023a), and both had been stored at 10°C (Table 6). These pH, a_w_ and temperature conditions resulted in predicted *ψ*–values of 1.04 and 1.02 (Table 9). So, both were very close to the growth boundary at 1.0, which means that even small uncertainties in the product characteristics or in the storage temperature could change the prediction from a no-growth response to a growth response. For these two specific observations, e.g., a change in pH–value to 5.13 or a change in storage temperature to 10.4°C, would change the *ψ*–values to become less than 1.0, moving these fail-dangerous no-growth responses to correct growth responses. These relatively small changes are within the uncertainties that would be expected for pH and temperature measurements when conducting challenge tests (Tables 2, 3). Therefore, care should be taken when disqualifying models exclusively based on data where uncertainties for intrinsic and extrinsic factors are not reported. Taking this into consideration, the results in Table 9 pointed at the updated Carlin et al. (2013) model for *panC* group II strain RIVM BC120 as the less biased and more accurate of the two models for predicting growth of *B. cereus s.l.* The model performed with an overall acceptable *B_f_* –value of 1.34, an *A_f_* –value of 1.57 close to being acceptable and with 70% correct predictions of growth/no-growth responses, classifying the model as generally fail-safe for foods containing starchy ingredients and stored at max 12°C.

**Table 9:** Evaluation of the two best performing of the updated *Bacillus cereus sensu lato* growth models using growth responses from both single and composite starchy foods as reported in the literature^a^ and growth responses in composite starchy foods generated in this study^b^.

Data	Carlin et al. (2013) ^c^							Zwietering et al. (1996) ^d^						
	*μ_max_* (h^−1^)			Growth/no-growth responses				*μ_max_* (h^−1^)			Growth/no-growth responses			
	n	Bias factor (*B_f_*)	Accuracy factor (*A_f_*)	n	% correct	% fail-safe	% fail-danger-ous	n	Bias factor (*B_f_*)	Accuracy factor (*A_f_*)	n	% correct	% fail-safe	% fail-danger-ous
Literature values (Table 6)	19	1.34	1.57	33	70	24	6^e^	21	1.68	1.82	33	70	30	0
Psychrotolerant thermotypes	12	1.40	1.59	13	92	8	0	12	1.97	1.99	13	92	8	0
Intermediary thermotypes	7	1.25	1.54	18	61	39	0	7	1.37	1.63	18	50	50	0
Single starchy foods	7	1.23	1.53	16	69	31	0	7	1.76	1.79	16	56	44	0
Composite foods	12	1.41	1.60	17	71	17	12^e^	14	1.64	1.83	17	82	18	0
Meat loaf	6	1.94	1.94	6	100	0	0	6	2.63	2.63	6	100	0	0
Other than meat loaf	13	1.14	1.43	27	63	30	7^e^	15	1.40	1.57	27	63	37	0
Composite foods this study (Table 4)	6	0.48	2.11	8	75	25	0	6	0.64	1.96	8	75	25	0

a Data in Table 6 were used.

b Data in Table 4 were used.

c Cardinal parameter model for *panC* group II strain RIVM BC120. *μ_opt_* was 1.41 h^−1^ and for the interaction term n in Equation 11 were 3 for temperature, 1 for pH and 3 for a_w_ (Table 8).

d Cardinal parameter model where *μ_opt_* was 2.00 h^−1^, expanded with an interaction term where n in Equation 11 were 3 for temperature, 3 for pH and 3 for a_w_ (Table 8).

e Represents two observations for pizza (ComBase, 2023a, 2023b) with *ψ*–values of 1.02 and 1.04, respectively.

As shown in Table 9, the *B_f_* – and *A_f_* –values, obtained using the updated Carlin et al. (2013) model for *panC* group II strain RIVM BC120, were better for the sub-dataset of challenge tests using intermediary thermotypes (*panC* groups IV and V) and for the sub-dataset of challenge tests involving single component starchy foods. For both sub-datasets, the evaluation was based on *n* = 7 growth responses (Table 9) but only for one growth response, these two sub-datasets overlapped, i.e., only one *μ_max_* observation was found for an intermediary thermotype (B4ac-1) in a single component starchy food (mashed potatoes from powder) (Mahakarnchanakul and Beuchat, 1999; Table 6), whereas six were found for intermediary thermotypes in composite starchy foods and six for psychrotolerant thermotypes in single component starchy foods. This indicated that *n* = 13 (*n* = 6 + 6 + 1) of the n = 19 *μ_max_* predictions, used in total for this evaluation, actually were less biased (*B_f_*, 1.14) and more accurate (*A_f_*, 1.43) than the overall averages (*B_f_*/*A_f_*, 1.34/1.57) (Table 9). Consequently, the remaining n = 6 (*n* = 19–13) *μ_max_* predictions represented the combination of psychrotolerant thermotypes in composite starchy foods. Applying this sub-dataset, which turned out to be the six challenges conducted for meat loaf, resulted in *B_f_* – and *A_f_* –values of 1.94, which were much higher than the overall averages (Table 9), indicating that growth was strongly over-predicted by the updated Carlin et al. (2013) model for *panC* group II strain RIVM BC120. This over-prediction was unexpected, as other studies reported faster growth of *B. cereus s.l.* when animal proteins were available in the substrate as compared to cereal proteins (Ellouze et al., 2021; Kang et al., 2010; Morita and Woodburn, 1977). The additional challenge tests (Table 4) using products with animal or vegetable proteins, and some of the strains as were used for updating the models, were, therefore, included in the present study to investigate this matter. For both of the updated models, *μ_max_* –values were strongly under-predicted with unacceptable *B_f_* –values below 0.7 (Table 9). Of the six observed growth responses, four were even below the lower acceptable *A_f_* –limit meaning that the observed *μ_max_* –values were more than 1.5-fold higher than predictions (results not shown). Interestingly, three of these four low-scoring *A_f_* challenges tests were from composite starchy foods rich in animal proteins and the remaining contained vegetable protein from split peas (Table 4). This confirms previous findings of growth rates of *B. cereus s.l.* in carbohydrate-rich foods being lower than in protein-rich foods, such as meat patties and tofu (Kang et al., 2010). Taken together this means that the updated Carlin et al. (2013) growth rate model for *panC* group II strain RIVM BC120 should not be used for composite protein-rich foods, as the growth rate might be under-predicted creating unsafe situations.

### 3.5 Predicting safe shelf lives using the best performing model

Knowing the time to reach a critical concentration of, e.g., 10^5^ cfu/g of *B. cereus s.l.* is an important input when deciding on the safe shelf-life for ready-to-eat or ready-to-cook chilled foods. The updated Carlin et al. (2013) model for *panC* group II strain RIVM BC120 (Table 9) can support this decision for foods consisting mainly of starchy ingredients, if the initial concentration (*N_0_*) and the lag time are known (Equation 3). With *μ_max_* –values predicted by the best performing model, lag times can be determined from the relative lag time (RLT) as Lag time = RLT × ln(2)/*μ_max_*. RLT is often a constant (Mellefont and Ross, 2003; Ross, 1999) and lag time has been calculated in this way for different pathogens and foods (Dalgaard and Mejlholm, 2019). In the present study, RLT was estimated using data from all the challenge tests showing growth in single starchy foods after a statistically significant lag time (*n* = 58) (Supplementary Tables S3, S4). The median RLT-value of 7.2 (95%-CI, 1.6–40) was selected as a representative value for the predicted examples (results not shown). Product examples were chosen based on known *N_0_* of *B. cereus s.l.*, i.e., concentrations measured close to the production time, as well as measured product characteristics (Table 10). The predictions in Table 10 demonstrated that keeping the storage temperature at max. 5°C was by far the most effective way of achieving a long safe shelf-life, i.e., at least 38 days. At this temperature, the time to reach the critical concentration of *B. cereus s.l.* was less affected by lowering pH or a_w_ than seen at the higher temperatures. Under storage at 8°C, the lowering of pH from 6.5 to 5.8 and a_w_ from 0.996 to 0.990 increased the time to reach the critical concentration of *B. cereus s.l*. with approx. 1.5–fold. The effect of having a low initial cell concentration can be seen when comparing rice with *N_0_* of 0.1 cfu/g to pasta with *N_0_* of 3 cfu/g. This showed that an *approx.* 10-fold lower *N_0_* resulted in 1, 2 and > 11 days longer time to reach the critical concentration of *B. cereus s.l.* at 10, 8 and 5°C, respectively (Table 10).

**Table 10:** Prediction of how many days it takes to reach a critical level of 10^5^ cfu/g of *Bacillus cereus s.l.* for selected cooked starchy foods when using the updated Carlin et al. (2013) model^a^ with and without a median relative lag time (RLT) of 7.2.

Cooked starchy food	pH	a_w_	*panC*-group identified	*N_0_* (cfu/g)	Time (days) to reach 10^5^ cfu/g when stored at					
					10°C		8 °C		5°C	
					RLT = 0	RLT = 7.2	RLT = 0	RLT = 7.2	RLT = 0	RLT = 7.2
Couscous (added 1% NaCl)	6.4^b^	0.989^b^	IV (III, VII)^b^	12^b^	4	6	7	10	38	>49^e^
Pasta (no added NaCl)	6.5^b^	0.996^b^	IV (III)^b^	3^b^	4	6	7	10	38	>49
Rice (no added NaCl)	6.4^b^	0.998^b^	IV (III)^b^	0.1^b^	5	7	9	12	>49	>49
Vegetable lasagna	5.8^c^	0.990^c^	V^d^	5^d^	5	7	11	16	>49	>49

a Cardinal parameter model for *panC* group II strain RIVM BC120. *μ_opt_* was 1.41 h^−1^ and for the interaction term n in Equation 11 were 3 for temperature, 1 for pH and 3 for a_w_ (Table 8).

b Determined as part of the present study. Isolates from the *panC* groups stated in brackets were also found in the samples.

c Assumed from meat lasagne (Table 5).

d Klein (2019).

e The maximum time frame of experiments used for evaluation of the model was 49 days.

## 4 Conclusion

The updated Carlin et al. (2013) cardinal parameter model for the *panC* group II strain RIVM BC120 performed better than available models when predicting both growth rate and growth/no-growth responses of *B. cereus s.l.* in single starchy foods at temperatures ≤12°C. The model was updated by adding a term for the inhibiting effect of interactions between temperature, pH and a_w_ as well as by product calibration of *μ_opt_*. The model performance was acceptable and on the safe side with *B_f_* – and *A_f_* –values of 1.34 and 1.57, respectively, for growth responses in starchy foods extracted from the scientific literature. The updated model is a useful tool for supporting food safety decisions regarding the growth potential of *B. cereus s.l.* in chilled ready-to-eat and ready-to-cook starchy foods. However, the updated Carlin et al. (2013) *μ_max_*–model performed poorly for composite protein-rich foods with *B_f_* – and *A_f_* –values of 0.48 and 2.11, respectively. The model should, therefore, not be used for composite starchy foods rich in animal and/or vegetable proteins, pointing to the need for the development of separate predictive models for such products to avoid under-predicting growth rate and creating unsafe situations.

## Data Availability

The original contributions presented in the study are included in the article/Supplementary material, further inquiries can be directed to the corresponding author.
