# Supplementary material for: Modeling and predicting growth and growth boundary of Bacillus cereus s.l. from phylogroups II, IV, V, and VI in starchy foods at or below 12°C
Source: Front Microbiol. 2025 Apr 30;16:1531014. doi: 10.3389/fmicb.2025.1531014 (PMC12080233; doi:10.3389/fmicb.2025.1531014)
Supplement: Supplementary file 1 [file Table_1.docx]

**Supplementary material**

| **Table S1.** Product characteristics, storage conditions and observed growth or no-growth within two weeks for preliminary challenge tests with *Bacillus cereus* *sensu* *lato* in selected starchy foods. | | | | | | | | | | | | | | |
| --- | --- | --- | --- | --- | --- | --- | --- | --- | --- | --- | --- | --- | --- | --- |
| Exp. no.^a^ | Cooked food | Isolate | Phylo- group^b^ | Measured characteristics^c^ | | | | | | Temp.^d^ (°C) | | Growth or no-growth within two weeks | | |
|  |  |  |  | pH | | WPS% | | aw | |  |  | A | B | C |
| Pre/E | Couscous | T101 | IV | 6.4 | (<0.1) | 1.8 | (0.07) | 0.990 | (<0.001) | 11.6 | (0.2) | Growth | Growth | Growth |
| Pre/E | Couscous | C246 | V | 6.1 | (<0.1) | 6.4 | (0.20) | 0.952 | (0.002) | 11.2 | (0.2) | No-growth | No-growth | No-growth |
| Pre/D | Mashed potato | RIVM BC120 | II | 6.2 | (<0.1) | 0.06 | (<0.01) | 0.999 | (<0.001) | 7.7 | (0.1) | Growth | Growth | Growth |
| Pre/E | Mashed potato | ADRIA-121 | VI | 6.2 | (<0.1) | 0.06 | (<0.01) | 0.999 | (<0.001) | 7.7 | (0.1) | Growth | Growth | Growth |
| Pre/E | Rice | NVH 0861-00 | II | 7.8 | (0.1) | 0.02 | (<0.01) | 0.999 | (<0.001) | 6.9 | (0.2) | No-growth | No-growth | No-growth |
| Pre/E | Rice | T101 | IV | 7.8 | (0.1) | 0.02 | (<0.01) | 0.999 | (<0.001) | 9.8 | (0.3) | Growth | Growth | Growth |
| Pre/D | Rice | ADRIA-121 | VI | 6.5 | (0.1) | 0.04 | (<0.01) | 0.999 | (<0.001) | 11.5 | (0.1) | Growth | Growth | Growth |
| ^a^ Pre stands for preliminary, D for data used for model development and E for data used for model evaluation.  ^b^ Phylogenitic (*panC*) group.  ^c^ Average of three samples with standard deviation in brackets.  ^d^ Average within the time frame of experiment with standard deviation in brackets. | | | | | | | | | | | | | | |

| **Table S2**. A screening design with temperature, pH and a_w_ as discrete numerical factors and phylogenetic (*panC*) groups as categorical factor established using the DOE function in SAS JMP Pro (RRID:SCR_022199) | | | | | | |
| --- | --- | --- | --- | --- | --- | --- |
| Exp.  no. | Starchy food | Phylogenetic (*panC*) group | Target values of | | | Used for |
|  |  |  | Temp. (°C) | pH | aw |  |
| 1 | Rice | II | 10 | 6 | 1 | Evaluation |
| 2 | Rice | II | 12 | 7 | 0.98 | Evaluation |
| 3 | Rice | VI | 7 | 5 | 0.97 | Evaluation |
| 4 | Rice | IV | 7 | 6 | 0.96 | Development |
| 5 | Pasta | VI | 12 | 6 | 1 | Evaluation |
| 6 | Couscous | IV | 12 | 6 | 0.98 | Development |
| 7 | Couscous | VI | 10 | 7 | 0.97 | Evaluation |
| 8 | Couscous | V | 7 | 5 | 0.96 | Evaluation |
| 9 | Couscous | II | 12 | 5 | 1 | Evaluation |
| 10 | Couscous | II | 7 | 6 | 0.98 | Development |
| 11 | Rice | V | 12 | 6 | 0.97 | Development |
| 12 | Couscous | V | 12 | 7 | 0.96 | Development |
| 13 | Rice | V | 7 | 7 | 1 | Evaluation |
| 14 | Pasta | V | 12 | 5 | 0.98 | Development |
| 15 | Pasta | VI | 7 | 6 | 0.97 | Evaluation |
| 16 | Pasta | VI | 10 | 6 | 0.96 | Evaluation |
| 17 | Pasta | IV | 12 | 6 | 1 | Development |
| 18 | Mashed potatoes | IV | 10 | 5 | 0.98 | Development |
| 19 | Pasta | IV | 12 | 5 | 0.97 | Evaluation |
| 20 | Bulgur | VI | 12 | 7 | 0.96 | Development |
| 21 | Mashed potatoes | V | 10 | 5 | 1 | Evaluation |
| 22 | Mashed potatoes | VI | 7 | 7 | 0.98 | Development |
| 23 | Bulgur | II | 12 | 7 | 0.97 | Evaluation |
| 24 | Bulgur | IV^a^ | 10 | 5 | 0.96 | Development |
| 25 | Rice | IV | 7 | 7 | 1 | Development |
| 26 | Rice | V | 10 | 7 | 0.98 | Evaluation |
| 27 | Rice | II | 10 | 5 | 0.97 | Development |
| 28 | Rice | II | 7 | 7 | 0.96 | Development |
| 29 | Pasta | VI | 7 | 5 | 1 | Evaluation |
| 30 | Mashed potatoes | IV | 10 | 6 | 0.98 | Evaluation |
| 31 | Pasta | V | 7 | 6 | 0.97 | Development |
| 32 | Pasta | II | 10 | 6 | 0.96 | Development |
| 33 | Rice | V | 10 | 7 | 1 | Development |
| 34 | Pasta | II | 7 | 5 | 0.98 | Development |
| 35 | Pasta | IV^a^ | 10 | 7 | 0.97 | Development |
| ^a^ Replaced with an isolate from phylogenetic (*panC*) group VI. | | | | | | |

| **Table S3.** Product characteristics, storage conditions and estimates of lag times (Lag) and max specific growth rates *(µ_max_*) for challenge tests used for the evaluation of literature models and for the subsequent calibration and expansion of *Bacillus cereus* *sensu* *lato* growth models. | | | | | | | | | | | | | | | | | |
| --- | --- | --- | --- | --- | --- | --- | --- | --- | --- | --- | --- | --- | --- | --- | --- | --- | --- |
| Exp. no. | Cooked food | Isolate | Phylo- group^a^ | Measured characteristics^b^ | | | | | | Temp.^c^ (°C) | | Sample | | | | | |
|  |  |  |  |  |  |  |  |  |  |  |  | A | | B | | C | |
|  |  |  |  | pH | | WPS% | | a_w_ | |  |  | Lag (h) | *µ_max_* (h^-1^) | Lag (h) | *µ_max_* (h^-1^) | Lag (h) | *µ_max_* (h^-1^) |
| 20 | Bulgur | ADRIA-I21 | VI | 6.8 | (<0.1) | 6.8 | (0.06) | 0.957 | (0.002) | 11.7 | (0.2) | 4.6 | 0.0388 | 31.7 | 0.0425 | 22.5 | 0.0415 |
| 24 | Bulgur | KBAB4 | VI | 5.1 | (<0.1) | 6.0 | (0.52) | 0.957 | (0.001) | 10.0 | (0.5) | NG^d^ | | NG | | NG | |
| 10 | Couscous | NVH 0861-00 | II | 6.4 | (0.1) | 3.3 | (0.02) | 0.983 | (0.001) | 6.9 | (0.2) | 225 | 0.0116 | 218 | 0.0288 | 216 | 0.0061 |
| 6 | Couscous | T101 | IV | 6.4 | (0.1) | 3.3 | (0.02) | 0.981 | (<0.001) | 11.5 | (0.1) | 236 | 0.1364 | 116 | 0.0613 | 149 | 0.1288 |
| 12 | Couscous | C246 | V | 6.4 | (<0.1) | 9.0 | (0.23) | 0.935 | (0.002) | 11.2 | (0.2) | NG | | NG | | NG | |
| Pre | Mashed potato | RIVM BC120 | II | 6.2 | (<0.1) | 0.06 | (<0.01) | 0.999 | (<0.001) | 7.7 | (0.1) | 44 | 0.0314 | 57.5 | 0.0379 | 70.2 | 0.0416 |
| 18 | Mashed potato | ATCC 14579 | IV | 5.9 | (<0.1) | 1.4 | (0.10) | 0.992 | (<0.001) | 10.8 | (0.6) | 109 | 0.0810 | 122 | 0.1037 | 50.0 | 0.0418 |
| 22 | Mashed potato | KBAB4 | VI | 6.2 | (<0.1) | 0.06 | (<0.01) | 0.999 | (<0.001) | 7.7 | (0.1) | 112 | 0.0671 | 104 | 0.0660 | 113 | 0.0658 |
| 32 | Pasta | RIVM BC120 | II | 6.1 | (<0.1) | 5.5 | (0.22) | 0.962 | (0.002) | 10.5 | (0.4) | 62.9 | 0.0338 | 49.1 | 0.0363 | 68.2 | 0.0390 |
| 34 | Pasta | NVH 0861-00 | II | 5.1 | (<0.1) | 3.0 | (0.16) | 0.978 | (0.001) | 7.5 | (0.4) | NG | | NG | | NG | |
| 17 | Pasta | C218 | IV | 6.0 | (<0.1) | 0.03 | (<0.01) | 0.995 | (0.001) | 11.0 | (0.1) | 59.0 | 0.0647 | 54.5 | 0.0624 | 57.0 | 0.0652 |
| 14 | Pasta | T126 | V | 4.8 | (<0.1) | 3.0 | (0.33) | 0.978 | (0.001) | 11.0 | (0.1) | NG | | NG | | NG | |
| 31 | Pasta | T126 | V | 6.1 | (<0.1) | 4.0 | (0.28) | 0.970 | (0.002) | 7.5 | (0.4) | NG | | NG | | NG | |
| 35 | Pasta | KBAB4 | VI | 6.5 | (<0.1) | 3.7 | (0.27) | 0.974 | (0.001) | 10.5 | (0.4) | -^e^ | 0.0731 | - | 0.0714 | - | 0.0762 |
| 28 | Rice | NVH 0861-00 | II | 6.6 | (<0.1) | 7.1 | (0.15) | 0.956 | (0.003) | 6.6 | (0.3) | NG | | NG | | NG | |
| 27 | Rice | NVH 0861-00 | II | 5.5 | (<0.1) | 5.7 | (0.1) | 0.964 | (0.001) | 10.0 | (0.5) | NG | | NG | | NG | |
| 11 | Rice | T101 | IV | 6.4 | (<0.1) | 3.4 | (0.06) | 0.981 | (<0.001) | 11.5 | (0.1) | 206 | 0.0590 | 214 | 0.1426 | 201 | 0.1170 |
| 25 | Rice | T101 | IV | 7.8 | (0.1) | 0.02 | (<0.01) | 0.999 | (<0.001) | 6.9 | (0.2) | NG | | NG | | NG | |
| 33 | Rice | C246 | V | 7.8 | (0.1) | 0.02 | (<0.01) | 0.999 | (<0.001) | 9.8 | (0.3) | - | 0.0475 | - | 0.0423 | - | 0.0444 |
| 4 | Rice | ADRIA-I21 | VI | 6.4 | (<0.1) | 6.4 | (0.20) | 0.960 | (0.001) | 6.0 | (0.1) | NG | | NG | | NG | |
| Pre | Rice | ADRIA-121 | VI | 6.5 | (0.1) | 0.04 | (<0.01) | 0.999 | (<0.001) | 11.5 | (0.1) | 20.7 | 0.2210 | 22.0 | 0.1915 | 16.1 | 0.1881 |
| ^a^ Phylogenetic (*panC*) group.  ^b^ Average of three samples with standard deviation in brackets.  ^c^ Average within the time frame of experiment with standard deviation in brackets.  ^d^ NG: no observed growth within the time frame of experiment 28 to 44 days.  ^e^ Lag time not statistically significant. | | | | | | | | | | | | | | | | | |

| **Table S4.** Product characteristics, storage conditions and estimates of lag times (Lag) and max specific growth rates (*µ_max_*) for challenge tests used for the evaluation of the calibrated and expanded *Bacillus cereus* *sensu* *lato* growth models. | | | | | | | | | | | | | | | | | |
| --- | --- | --- | --- | --- | --- | --- | --- | --- | --- | --- | --- | --- | --- | --- | --- | --- | --- |
| Exp. no. | Cooked food | Isolate | Phylo- group^a^ | Measured characteristics^b^ | | | | | | Temp.^c^ (°C) | | Sample | | | | | |
|  |  |  |  |  |  |  |  |  |  |  |  | A | | B | | C | |
|  |  |  |  | pH | | WPS% | | a_w_ | |  |  | Lag (h) | *µ_max_* (h^-1^) | Lag (h) | *µ_max_* (h^-1^) | Lag (h) | *µ_max_* (h^-1^) |
| 23 | Bulgur | RIVM BC120 | II | 6.8 | (<0.1) | 5.0 | (0.23) | 0.967 | (0.006) | 11.7 | (0.2) | 61.2 | 0.0846 | 46.8 | 0.0797 | 59.5 | 0.0865 |
| 9 | Couscous | NVH 0861-00 | II | 5.4 | (<0.1) | 0.3 | (<0.01) | 0.992 | (0.002) | 11.2 | (0.2) | 72.5 | 0.0667 | 85.5 | 0.0745 | 86.0 | 0.0747 |
| Pre | Couscous | T101 | IV | 6.4 | (<0.1) | 1.8 | (0.07) | 0.990 | (<0.001) | 11.6 | (0.2) | -^d^ | 0.1386 | - | 0.1231 | - | 0.1209 |
| Pre | Couscous | C246 | V | 6.1 | (<0.1) | 6.4 | (0.20) | 0.952 | (0.002) | 11.2 | (0.2) | NG^e^ | | NG | | NG | |
| 8 | Couscous | C246 | V | 5.3 | (<0.1) | 8.0 | (0.02) | 0.944 | (0.001) | 6.6 | (0.2) | NG | | NG | | NG | |
| 7 | Couscous | ADRIA-I21 | VI | 6.4 | (<0.1) | 6.9 | (0.3) | 0.951 | (0.001) | 9.7 | (0.5) | NG | | NG | | NG | |
| 21 | Mashed potato | T126 | V | 5.9 | (<0.1) | 2.2 | (0.08) | 0.987 | (<0.001) | 10.8 | (0.6) | 35.6 | 0.0793 | 43.0 | 0.0820 | 41.5 | 0.0803 |
| 30 | Mashed potato | C218 | IV | 5.9 | (<0.1) | 1.6 | (0.03) | 0.991 | (<0.001) | 10.8 | (0.6) | NG | | NG | | NG | |
| Pre | Mashed potato | ADRIA-I21 | VI | 6.1 | (<0.1) | 0.06 | (<0.01) | 0.999 | (<0.001) | 7.7 | (0.1) | 248 | 0.0653 | 214 | 0.0491 | 231 | 0.0594 |
| 19 | Pasta | ATCC 14579 | IV | 4.8 | (<0.1) | 4.6 | (0.13) | 0.971 | (0.003) | 11.0 | (0.1) | NG | | NG | | NG | |
| 5 | Pasta | ADRIA-I21 | VI | 6.6 | (<0.1) | 0.00 | (<0.01) | 0.999 | (<0.001) | 11.5 | (0.1) | 43.6 | 0.1377 | 43.6 | 0.1189 | 35.6 | 0.0766 |
| 16 | Pasta | KBAB4 | VI | 6.0 | (<0.1) | 6.2 | (0.12) | 0.959 | (0.015) | 10.8 | (0.6) | NG | | NG | | NG | |
| 29 | Pasta | ADRIA-I21 | VI | 5.0 | (<0.1) | 0.06 | (>0.01) | 0.996 | (<0.001) | 7.5 | (0.4) | NG | | NG | | NG | |
| 15 | Pasta | KBAB4 | VI | 6.0 | (<0.1) | 4.7 | (0.07) | 0.970 | (0.002) | 6.7 | (0.2) | NG | | NG | | 729 | 0.0096 |
| 2 | Rice | NVH 0861-00 | II | 6.4 | (<0.1) | 3.4 | (0.09) | 0.978 | (<0.001) | 11.5 | (0.1) | 53.3 | 0.0954 | 54.3 | 0.0970 | 45.0 | 0.0866 |
| 1 | Rice | NVH 0861-00 | II | 7.8 | (0.1) | 0.02 | (<0.01) | 0.999 | (<0.001) | 9.8 | (0.3) | 19.4 | 0.0778 | 20.5 | 0.0780 | 20.5 | 0.0844 |
| Pre | Rice | NVH 0861-00 | II | 7.8 | (0.1) | 0.02 | (<0.01) | 0.999 | (<0.001) | 6.9 | (0.2) | NG | | NG | | NG | |
| Pre | Rice | T101 | IV | 7.8 | (0.1) | 0.02 | (<0.01) | 0.999 | (<0.001) | 9.8 | (0.3) | 98.0 | 0.0582 | 114 | 0.0606 | 123 | 0.0701 |
| 26 | Rice | T126 | V | 6.6 | (<0.1) | 3.7 | (0.08) | 0.979 | (0.001) | 10.0 | (0.5) | 57.6 | 0.0791 | 68.9 | 0.0942 | 81.9 | 0.1018 |
| 13 | Rice | C246 | V | 7.8 | (0.1) | 0.02 | (<0.01) | 0.999 | (<0.001) | 6.9 | (0.2) | NG | | NG | | NG | |
| 3 | Rice | ADRIA-I21 | VI | 6.4 | (<0.1) | 4.8 | (0.04) | 0.971 | (0.001) | 6.0 | (0.1) | NG | | NG | | NG | |
| ^a^ Phylogenetic (*panC*) group.  ^b^ Average of three samples with standard deviation in brackets.  ^c^ Average within the time frame of experiment with standard deviation in brackets.  ^d^ Lag time not statistically significant.  ^e^ NG: no observed growth within the time frame of experiment 28 to 44 days. | | | | | | | | | | | | | | | | | |
